# The efficacy and safety of intravenous tranexamic acid on blood loss during total ankle replacement: a retrospective study

**DOI:** 10.1038/s41598-022-13861-3

**Published:** 2022-06-09

**Authors:** Gang Tan, Li wei Xie, Shi Jiu Yi, Yu Chen, Xi Liu, Hui Zhang

**Affiliations:** 1 Department of Orthopaedics, West China Hospital of Sichuan University, No.37 of Guoxue lane, Wuhou District, Chengdu, 610041 Sichuan China; 2 Department of Orthopaedics, West China School of Public Health and West China Fourth Hospital, Sichuan University, Chengdu, Sichuan China; 3 Department of Pediatric Orthopaedics, Sichuan Provincial Orthopaedics Hospital, Chengdu, Sichuan China

**Keywords:** Diseases, Medical research

## Abstract

Only a few of studies have reported whether Tranexamic acid (TXA) has the same benefits during total ankle replacement as hip and knee replacements. In our study, we hypothesized that TXA was effective in reducing the perioperative blood loss without increasing the risk of symptomatic venous thromboembolism of patients during total ankle replacement. We retrospectively reviewed 71 patients who underwent total ankle replacement at the foot and ankle surgery center of our institution between January 2017 and May 2021. Patients were divided into two groups according to whether they received intravenous tranexamic acid or not. Patients who received intravenous TXA showed significantly lower estimated intraoperative blood loss, hidden blood loss and total blood loss. The early AOFAS score and ROM at the first month follow up of TXA group were better than the NO-TXA group and the incidence of early wound infection and poor healing (< 1 month postoperative) was significant lower than NO-TXA group. Use of TXA was not associated with significant changes in the incidence of postoperative symptomatic venous thromboembolism. We conclude that intravenous TXA can safely and effectively reduce perioperative blood loss and the incidence of early wound infection and poor healing in total ankle replacement, without increasing the risk of symptomatic venous thromboembolism.The application of TXA following total ankle replacement helped improve ankle function and patient quality of life during the early stage.

## Introduction

The efficacy and safety of tranexamic acid (TXA) in hip and knee replacement has been extensively studied in the orthopedic department of our hospital^1–5^, and studies have shown that TXA can obviously reduce blood loss and transfusion rate during the perioperative period in total hip and total knee replacement. It has also been reported that the use of TXA in shoulder replacement can reduce perioperative blood loss and complications^6,7^, and the use of TXA in trauma orthopedics and spinal fusion can also significantly reduce perioperative blood loss and related complications^8–10^. However, there are few reports on the application of TXA in total ankle replacement (TAR), and the conclusions of the two existing studies are completely opposite. In a retrospective study by Nodzo et al., they analyzed the perioperative blood loss and complication rate of patients who received TXA and those who did not use TXA during TAR, and pointed out that the use of 1 g TXA before TAR can significantly reduce the perioperative blood loss and the incidence of wound complications compared with patients who did not receive TXA^11^. However, Steinmet et al. analyzed perioperative blood loss and the incidence of related complications in patients receiving TXA or not in a retrospective study, concluding that TXA could not significantly reduce the perioperative blood loss and complication rate of TAR^12^.

In our center, as long as patients have no contraindications to TXA, they have administered TXA during TAR routinely after 2018. The administration ways contain intravenous, oral, topical, and the dosage include single dose, multiple boluses, high initial-dose. Our team retrospectively analyzed the patients who underwent total ankle replacement in the foot and ankle surgery center of our hospital from January 2017 and May 2021, and divided the patients into two groups who did not use TXA as NO-TXA group and the patients who used TXA as TXA group. We hypothesized that TXA was effective in reducing the perioperative blood loss without increasing the risk of symptomatic venous thromboembolism of patients during total ankle replacement.

## Methods

This is a retrospective study. Medical records were screened for patients undergoing total ankle replacement between January 2017 and May 2021 at the foot and ankle surgery center of our institution. Only medical records from patients that met all the inclusion criteria and none of the exclusion criteria were assessed in this study. In the orthopedics department of our institution, TXA was initially used in total hip and knee arthroplasty. When its safety and effectiveness were confirmed, it began to be vigorously promoted and applied in spinal surgery, trauma, and sports medicine. Moreover, the application of TXA in TAR was the latest. Therefore, many patients who accepted TAR did not use TXA during the perioperative period. All total ankle replacement operations were performed by the same senior ankle surgeon (Prof. H.Z.) and also followed the same surgeon (Prof. H.Z.) at the same orthopedics Clinic. The minimum follow up for the patients was 6 months after operation (average, 28 months; range, 6–42 months). All patients were cared by the same group of nurses. This clinical study protocol and proposal were approved by the Institutional Review Board of West China Hospital of Sichuan university and informed consent was obtained from all the participants who were divided into two groups according to whether they received intravenous TXA or not. The patient profiles of the 2 groups were reviewed respectively. For all patients, the follow-up outcomes at 2 weeks, 1 month, 3 months, 6 months and 12 months after the operation were recorded, respectively. When the postoperative follow-up lasted more than 12 months, they were followed up once a year. This study was performed in accordance with the 'Declaration of Helsinki'.

The results included estimated intraoperative blood loss, hemoglobin (Hb) of preoperative and postoperative 72 h, hematocrit (HCT) of preoperative and postoperative 72 h, hidden blood loss, total blood loss, the incidence of symptomatic venous thromboembolism (VTE), the occurrence of early wound complications such as infection and poor healing (< 1 month postoperative), the American Orthopedic Foot and Ankle Society (AOFAS) score, and the ankle range of motion (ROM, dorsifle- xion and plantar flexion) at every follow-up. Clinical measurements were performed by two individuals (G.T. and Y.C.).

### Inclusion criteria

 (i) the patients underwent TAR in the Department of orthopedics at our hospital between January 2017 and May 2021; (ii) patients were injected intravenous TXA (Chongqing Lummy Pharmaceutical Co., Ltd., Chongqing, China) 5 to 10 min before skin incision as an intravenous preoperative dose (15 mg/kg) in all cases, and it was administered again intravenously at the point when the surgery exceeded 2 h and all patients no longer received TXA after operation; (iii) patients were not treated with TXA; and (iv) the related outcomes of patients were complete in the medical records.

### Exclusion criteria

 (i) patient underwent ankle joint revision surgery; (ii) contraindications to TXA, including renal failure (serum creatinine > 200 mmol/L, creatinine clearance < 50 ml/min, or dialysis), lifelong use of anticoagulants, severe coronary artery disease or diffuse intravascular coagulation, allergy to TXA, and thrombolytic therapy or previous thrombolysis within 1 year before the operation events (myocardial infarction, cerebrovascular accident, deep vein thrombosis, and pulmonary embolism); (iii) TXA was used postoperatively.

### Perioperative management

 (i) Before the operation, all patients underwent routine systemic physical examinations to exclude hidden infection, and to confirm that the erythrocyte sedimentation rate and C-reactive protein levels were normal, and the previous complicated internal diseases such as hypertension, diabetes, and rheumatoid disease were well controlled. (ii) Three days before the operation, the patients were instructed in terms of cough, expectoration, and leg lift exercises for post-surgery care. (iii) All patients were administered 2 g of Cefuroxime sodium half an hour before operation and every 12 h within the first 24 h post-operation to prevent surgical site infection. If the operation time exceeds 3 h or the amount of bleeding is more than 800 ml, the same dose of Cefuroxime sodium has been added again during the operation. (iv) Tourniquets were used to control bleeding during the operation and all patients underwent an anterior approach incision; (v) After the procedure was completed, a drainage tube was placed before closing the incision. When the drainage volume in a single day post-operation was less than 50 ml or on the third day after operation, the drainage tube was removed; (vi) Postoperative analgesia was administered in accordance with the perioperative pain management plan, and opioids were used for temporary auxiliary analgesia if necessary; (vii) After the operation, the patients were placed in a head-low and foot-high position. Post-operative ankle joint functional exercises of all patients were guided and supervised by the same rehabilitation physician who carried out the same rehabilitation training plan at first and adjust individually according to the actual situation during the training process. Briefly, within 3 weeks post-operation, patients should exert rehabilitation training in bed. For example, straight leg lifting exercise and respiratory function exercise such as expectoration were encouraged according to the preoperative guidance from the second day after the operation to prevent venous thromboembolism (VTE) , and patients need to wear short leg cast to keep the ankle in a neutral position for 24 h every day. After 3 weeks post-operation, the cast was then removed to allow patients starting ankle flexion and extension functional exercise, and gradually done incomplete weight-bearing exercise with inflatable boots (starting from stepping on the ground with a weight of 5 kg, increasing 5 kg every 5 days until full weight-bearing functional exercise). When the bearing weight increased to half of the body weight, the patient began full weight-bearing function exercise; (viii) Low-molecular weight heparin (0.2 ml) was administered for all patients at 12 h after surgery and then 0.4 ml every 24 h or 10 mg of Rivaroxaban (Xarelto; Bayer, Leverkusen, Germany) was administered orally once a day for 35 days to prevent VTE if no bleeding events occurred. Doppler ultrasound was used routinely to detect deep venous thrombosis (DVT) at the time of discharge and at 30-day follow-up, or when there was a clinically suspected DVT; (ix) Indications for allogeneic blood transfusion were Hb < 70 g/L or 70 g/L < Hb < 100 g/L in the presence of dizziness, palpitations, chest tightness, weakness, and other anemia symptoms; (x) General anesthesia was used for all patients.

### Outcomes measurement

#### Demographic data

Demographic data included age, gender, height, weight, comorbidities, and body mass index (BMI) was calculated in kg/m^2^ based on height and weight.

#### Blood loss

The methods to calculate estimated intraoperative blood loss, visible blood loss and hidden blood loss as previously described^13,14^.

Preoperative HCT was defined as the HCT within 1 day before surgery.

Estimated intraoperative blood loss = the volume of liquid in the negative pressure aspirator-the volume of flushing saline + net weight gain of the gauze. The preoperative weight of the dry gauze with exact specification was known. Thereafter, the circulating nurse weighed the used gauze with an electronic scale after the surgery. Finally, we got the net weight gain of the gauze.

Visible blood loss is the sum of estimated intraoperative blood loss and postoperative drainage volume.

Hidden blood loss was calculated as follows. First, the patient’s blood volume (PBV) was calculated according to the formula^14^:

PBV (mL) = [k1 × height (m) 3 + k2 × weight (kg) + k3] × 1000, where k1 = 0.3669, k2 = 0.03219, and k3 = 0.6041 for male patients, whereas k1 = 0.3561, k2 = 0.03308, and k3 = 0.1833 for female patients. Next, the Gross equation was used to calculate hidden blood loss based on HCT:

Hidden blood loss = PBV × 2 ×  (preoperative HCT—HCT of postoperative 72 h)/ (preoperative HCT + HCT of postoperative 72 h) + autologous blood transfusion volume + allogeneic blood transfusion volume—visible blood loss.

Total blood loss = Visible blood loss + Hidden blood loss.

#### Postoperative symptomatic VTE

The safety outcomes were represented by postoperative symptomatic VTE including symptomatic deep vein thrombosis (DVT) and symptomatic pulmonary embolism (PE) that caused clinical symptoms, such as lower extremity pain, swelling, circumference change, positive homans sign, chest tightness, chest pain, hemoptysis, and decreased oxygen saturation during hospitalization.When these symptoms occured, the ultrasonography of double lower limbs, the blood coagulation indicator, blood gas analysis tests and CT pulmonary angiography (CTPA) were performed partially or even completely. If the results were positive, it were defined as symptomatic DVT or even symptomatic PE.

#### Infection

The diagnosis of infection was assessed according to the hospital infection standard formulated by the Center for Disease Control and Prevention in 1992 whose symptoms and signs contained fever, local redness, exudate, and its characteristics recorded in the medical history within 30 days after the operation, combined with the changes of blood inflammatory indexes such as the erythrocyte sedimentation rate and C-reactive protein, as well as secretion bacterial smear and culture results^11^.

### Statistical analysis

Statistics were calculated using Statistical Package for the social sciences version 25 (IBM SPSS Corp.; Armonk, NY, USA).All the data are expressed as mean and standard deviation, unless otherwise indicated. Because the demographic characteristics of the patients, such as age and BMI, in both groups were normally distributed, the diferences in continuous data between both groups were analyzed using Student's t-test, and categorical data (i.e.Sex) were analyzed using Pearson's chi-square test or Fisher's exact probability test. The postoperative outcomes data were not normally distributed and were analyzed using the Mann–Whitney U test. Differences were considered statistically significant at a *P* value of < 0.05.

### Ethics approval and consent to participate

This clinical study protocol and proposal were approved by the Institutional Review Board of West China Hospital of Sichuan university and informed consent was obtained from all the participants.

## Results

### General information and demographic data

A total of 71 patients met the selection criteria and were included in the study.The prosthesis which was adopted for TAR in this study was INBONE II (Wright Medical, Arlington, TN). Of the 71 patients, 33 did not receive TXA (represent as No-TXA) treatment and 38 received TXA (represent as TXA) treatment. The general demographic information of the patients are shown in Table 1.

**Table 1:** Demographic characteristic of all patients.

Variable	TXA (n = 38)	No-TXA (n = 33)	*P* Value
Age (years)	65.84 ± 5.63	62.66 ± 6.92	0.642
Sex (male/female)	17/21	16/17	0.814
BMI (kg/m^2^)	25.73 ± 1.96	26.49 ± 2.14	0.852
**Ankle diagnosis (n, %)**			
Osteoarthritis	24 (63.16%)	21 (63.64%)	
Post-traumatic arthritis	11 (28.95%)	9 (27.27%)	
Other	3 (8.89%)	3 (9.09%)	
**Comorbidities (n, %)**			
Diabetes	6 (15.79%)	6 (18.18%)	0.788
Smoking	6 (15.79%)	7 (21.21%)	0.759
Alcoholism	7 (18.42%)	8 (24.24%)	0.574
Gout	1 (2.63%)	2 (6.06%)	0.594
Glucocorticoid usage	2 (5.26%)	1 (3.03%)	0.641
COPD	2 (5.26%)	2 (6.06%)	0.884
**Side (n, %)**			
Right	21 (55.26%)	19 (57.58%)	
Left	17 (44.74%)	14 (42.42%)	

*COPD* chronic obstructive pulmonary disease.

The average duration of surgery and the details of associated procedures between the two groups are shown in Table 2.

**Table 2:** Details of average duration of surgery and associated procedures between the two groups.

Variable	TXA (n = 38)	No-TXA (n = 33)	*P* Value
Duration of surgery (min)	223.57 ± 42.15	205.92 ± 45.02	0.260
Lateral ligament reconstruction (n, %)	24 (63.16%)	21 (63.64%)	0.967
Medial collateral ligament release (n, %)	26 (68.42%)	21 (63.64%)	0.671
Subtalar fusion (n, %)	2 (5.26%)	1 (3.03%)	0.641
Achilles tendon lengthening (n, %)	18 (47.37%)	19 (57.58%)	0.316
Calcaneus osteotomy (n, %)	2 (5.26%)	2 (6.06%)	0.884
Fibular osteotomy (n, %)	2 (5.26%)	3 (9.09%)	0.530
Tibial osteotomy (n, %)	2 (5.26%)	3 (9.09%)	0.530
Medial malleolus osteotomy (n, %)	1 (2.63%)	2 (6.06%)	0.474
Tendon transposition (n, %)	3 (7.89%)	1 (3.03%)	0.375

### Effectiveness

No statistically significant difference of preoperative level of Hb and HCT were detected between the two groups. However, a significant difference of postoperative 72 h level of Hb and HCT were detected. The TXA group showed lower estimated intraoperative blood loss, hidden blood loss, total blood loss and Hb decrease than NO-TXA group. There were no blood transfusion events in TXA group after surgery while one case accepted blood transfusion in the NO-TXA group. The results are shown in Table 3.

**Table 3:** Blood loss of all patients.

Variable	TXA (n = 38)	No-TXA (n = 33)	*P* Value
Preoperative Hb (g/L)	135.74 ± 11.89	140.06 ± 10.85	0.114
Postoperative Hb (g/L)	123.26 ± 12.80	114.12 ± 6.14	0.000
Preoperative HCT (%)	40.71 ± 3.65	40.64 ± 2.78	0.924
Postoperative HCT (%)	35.76 ± 3.82	32.79 ± 1.99	0.000
Change in Hb (g/L)	15.50 ± 2.46	23.15 ± 4.31	0.000
Hidden blood loss (mL)	296.08 ± 76.30	399.68 ± 154.25	0.015
total blood loss (mL)	522.27 ± 164.28	650.94 ± 169.96	0.027
Estimated blood loss (mL)	89.74 ± 9.72	99.24 ± 8.85	0.000
Need for transfusion (n)	0 (0%)	1 (3.03%)	

**Figure 1:**
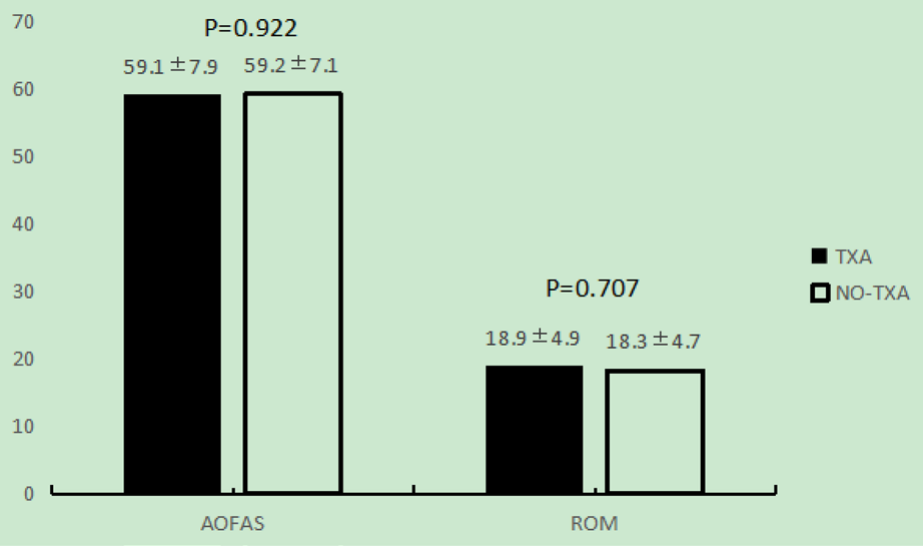
The average AOFAS score and ROM of preoperation between the two groups. The TXA group is shown as black bars and the NO-TXA group is shown as white bars.

The average AOFAS score and ROM of preoperation between the two groups have no significant difference (Fig. 1).

**Figure 2:**
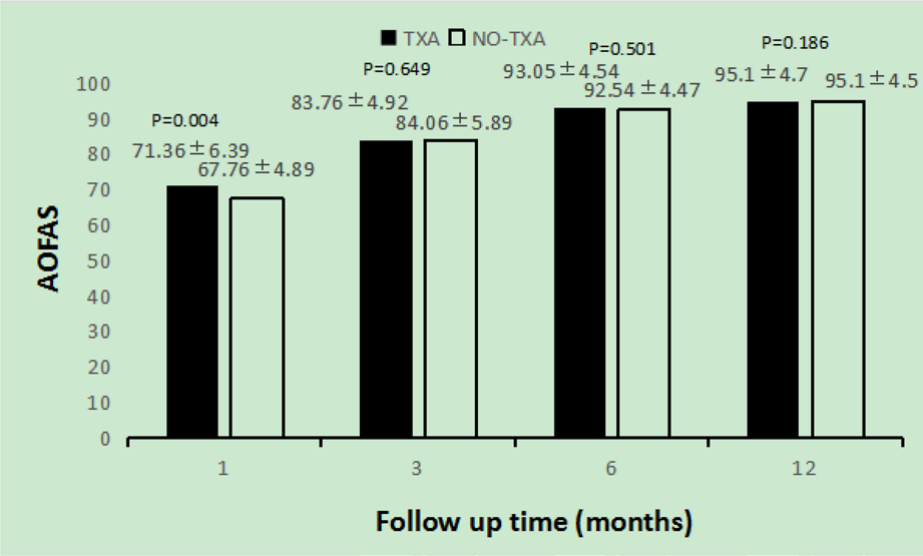
The AOFAS score of both groups at each follow up. The TXA group is shown as black bars and the NO-TXA group is shown as white bars.

**Figure 3:**
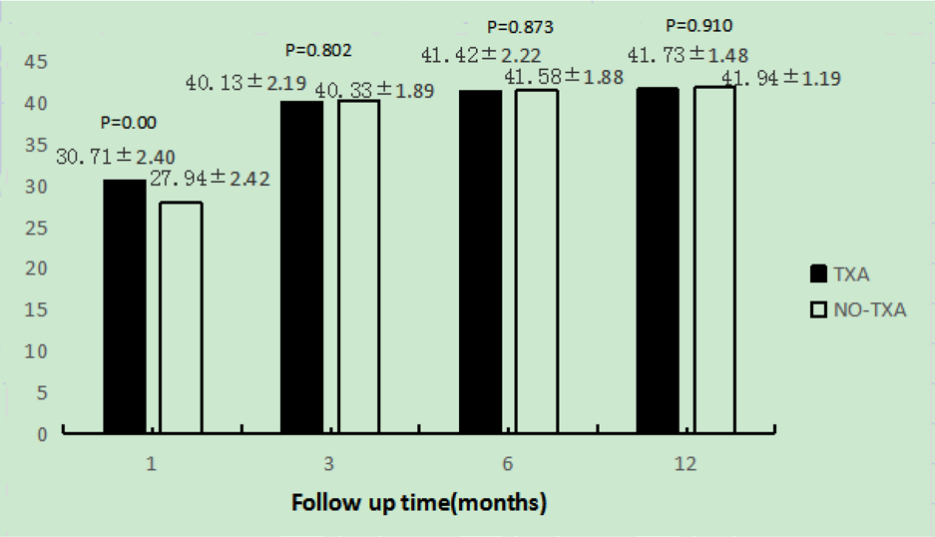
The ROM of both groups at each follow up. The TXA group is shown as black bars and the NO-TXA group is shown as white bars.

Four weeks after the operation, the average AOFAS score in the TXA group were significantly higher than those in the NO-TXA group. However, from 3 months to 1 year after the operation, there was no statistically significant difference in the average AOFAS score between the two groups (Fig. 2). Similarly, the ROM in the TXA group were significantly higher than those in the NO-TXA, and from 3 months to 1 year after the operation, there was no statistically significant difference in the average AOFAS score between the two groups (Fig. 3).

### Safety

There were 6 cases in the NO-TXA group in which incisions occurred poor wound healing. 2 patient underwent secondary debridement and suturing, and the other four cases were wound-healed after enhanced dressing changes. Only one case in the TXA group had poor wound healing, and the incision was healed after enhanced dressing changes. All seven patients with postoperative wound complications required oral antibiotics for at least 4 weeks along with local incision care.

No serious adverse reactions, or symptomatic venous thromboembolism (VTE) occurred in either group but one case in the No-TXA group was detected intermuscular vein thrombosis on the operative side at the 5th day after operation by Doppler examination which was an asymptomatic incomplete thrombosis, and no special treatment was administered. In the No-TXA group, one case of cerebrovascular accident occurred, which was a lacunar cerebral infarction, and the symptoms disappeared after neurological treatment, while no cardio-cerebrovascular accident occurred in the TXA group. The detailed complications following TAR shown in Table 4.

**Table 4:** Complications following TAR.

Variable	TXA (n = 38)	No TXA (n = 33)	*P* Value
Early wound infection and poor healing (< 1 month postoperative)	1	6	0.044
Cardiovascular and cerebrovascular accident	0	1	0.465
Late deep infection (> 3 mo postoperative)	0	0	
Symptomatic VTE	0	0	

## Discussion

TAR has become an effective choice for the treatment of end-stage ankle arthritis^15–18^. The perioperative blood loss in primary TAR includes overt blood loss containing intraoperative estimated blood loss and postoperative drainage, and hidden blood loss, such as extravasation into the tissue, residual blood in the joint, and loss due to hemolysis, accounts for even 50% of the total blood loss^19^. TXA has been widely used in other orthopaedic surgeries and has been proven to significantly reduce perioperative blood loss and reduce the patient blood transfusion rate^20–23^. The activation of fibrinolysis in patients after surgery is an important factor that causes intraoperative and postoperative blood loss^24^ which was activated at the beginning of the operation and 6 to 12 h is the most obvious^25^. As a fibrinolytic inhibitor, TXA is mainly used for 0 to 24 h after operation and its safety and effectiveness have been proven^26–28^. A large number of previous studies have shown that a single preoperative use of TXA can reduce the amount of intraoperative and postoperative blood loss, but it does not increase the incidence of deep vein thrombosis in patients^6,29^.

There are a few of studies concerning the application of TXA in TAR. Nodzo et al.^11^ retrospectively analyzed perioperative blood loss, incision complication rate, and blood transfusion rate in patients who received TAR treatment and they concluded that comparing with the NO-TXA group, a single dose of 1 g TXA administered intravenously before incision can significantly reduce perioperative blood loss and the incidence of wound complications. However, in a study by Steinmetz et al.^12^ they also retrospectively analyzed the perioperative blood loss, blood transfusion rate and incision complication rate of patients who undertaken TAR. They found and concluded that whether or not tranexamic acid was used, there was no significant difference in intraoperative blood loss, postoperative hemoglobin change, wound complication rate and blood transfusion rate, they believe that the possible reason is that all the operations in their study were performed by two senior foot and ankle surgeons, and the tourniquet was stopped after the prosthesis was implanted, and the hemostasis was performed before the closure of the anterior ankle wound. In addition, they believe that although the soft tissue of the anterior ankle is thin and the risk of wound disruption and other complications is very high when a local hematoma is formed in front of the ankle after TAR, but if the operator can pay careful attention to hemostasis during the operation, the risk of incision related complications can be well controlled. They also analyzed the reasons why tranexamic acid could not effectively reduce perioperative blood loss, possibly because the study was retrospective analysis, and not all patients had blood routine examination results on the first day after operation.

The main finding of our study is that usage of TXA can reduce the perioperative blood loss of patients accepted TAR, especially the estimated blood loss, hidden blood loss and total blood loss, and also can reduce the incidence of early incision complications (early wound infection and poor healing), but without increasing the risk of VTE. In our study, there was no statistical difference detected in the demographic data between the two groups of patients. All patients who underwent TAR were operated by the same professor, including the incision suture. However, it is found that comparing with the patients in the NO-TXA group, the intraoperative and postoperative blood loss and the the incidence of early incision complications of the TXA group were reduced, and statistically significant differences were detected, which is similar to the results of Nodzo et al.'s study. We also found that the early AOFAS score and ROM of the TXA group were better than the NO-TXA group, and statistically significant differences were detected. However, there was no statistically significant difference in the average AOFAS score between the two groups three months, six months and twelve months, the possible reason may be TXA can reduce the level of C-reactive protein and erythrocyte sedimentation rate during the early stage postoperation^4^.

In the above mentioned two literature reports about the efficiency of TXA on TAR, the authors chosen the hematological examination results on the first day after surgery as the research index. However, according to a large number of documents^1–5^, the calculation of hidden blood loss is based on the HCT of preoperative and postoperative 72 h. Therefore, we believe that as Steinmetz, et al. said when analyzing the limitations in their study, they collected the hematological at the first day postoperative which may be biased. In addition, they did not calculate the hidden blood loss. Although the use of tourniquet during TAR can effectively control the intraoperative bleeding, the hidden blood loss is one of the important factors of perioperative management of orthopedic surgery, and the hidden blood loss will affect the postoperative recovery of patients. In our study, the estimated blood loss, hidden blood loss, and total blood loss in the TXA group were lower than those in the NO-TXA group, indicating that TXA can significantly reduce perioperative blood loss in TAR patients.

Our study does have two major limitations that need to be pointed out. The first one is that the retrospective nature of its design which allows for potential selection bias. In the future work, prospective randomized controlled trails can effectively solve this limitation. The second limitation is that the relatively small sample size, although our study shows that TXA can effectively reduce the perioperative blood loss and the incidence of incision complications in TAR, larger sample sizes would be more beneficial to detect clinical differences.

## Conclusion

Based on our findings, TXA can safely and effectively reduce perioperative blood loss and the incidence of early wound infection and poor healing in TAR as total knee arthroplasty and total hip arthroplasty, but without increasing the risk of symptomatic venous thromboembolism. Meanwhile, the application of TXA following TAR helped improve ankle function and patient quality of life during the early stage.

## Data Availability

The datasets used and analyzed during the current study are available from the corresponding authors on reasonable request.
